# Origins and Evolution of WUSCHEL-Related Homeobox Protein Family in Plant Kingdom

**DOI:** 10.1155/2014/534140

**Published:** 2014-01-06

**Authors:** Gaibin Lian, Zhiwen Ding, Qin Wang, Dabing Zhang, Jie Xu

**Affiliations:** ^1^School of Life Sciences and Biotechnology, Shanghai Jiao Tong University, Shanghai 200240, China; ^2^Department of Physics, Shanghai Jiao Tong University, Shanghai 200240, China; ^3^Institutes of Biomedical Sciences, Fudan University, Shanghai 200032, China

## Abstract

WUSCHEL-related homeobox (WOX) is a large group of transcription factors specifically found in plants. WOX members contain the conserved homeodomain essential for plant development by regulating cell division and differentiation. However, the evolutionary relationship of WOX members in plant kingdom remains to be elucidated. In this study, we searched 350 WOX members from 50 species in plant kingdom. Linkage analysis of WOX protein sequences demonstrated that amino acid residues 141–145 and 153–160 located in the homeodomain are possibly associated with the function of WOXs during the evolution. These 350 members were grouped into 3 clades: the first clade represents the conservative WOXs from the lower plant algae to higher plants; the second clade has the members from vascular plant species; the third clade has the members only from spermatophyte species. Furthermore, among the members of *Arabidopsis thaliana* and *Oryza sativa*, we observed ubiquitous expression of genes in the first clade and the diversified expression pattern of *WOX* genes in distinct organs in the second clade and the third clade. This work provides insight into the origin and evolutionary process of WOXs, facilitating their functional investigations in the future.

## 1. Introduction

Homeobox genes encode transcript factors containing a classic DNA binding domain (called homeodomain) with about 60 amino acids (aa), which forms three helixes in space. The homeobox gene was first identified in *Drosophila *[1, 2]. Subsequently, more homeobox members have been reported in most eukaryotes [3]. WOX (WUSCHEL-related homeobox) is the member of ZIP superfamily belonging to homeobox proteins family [4].

In *Arabidopsis thaliana*, *WUSCHEL *(*WUS*) is essential in maintaining shoot apical meristem (SAM); *WUS *mutants display aborted SAM maintenance during embryonic and later developmental stages [5, 6]. The expression of *AtWOX2* is detectable in the egg cell and zygote, and *AtWOX2 *functions in zygotic apical cell development [7]. Transcripts of *AtWOX3 *are observed in the peripheral area of SAM, and *AtWOX3 *is implicated in forming horizontal regions of vegetative and floral organs [8].* AtWOX5*, a close homolog of *AtWUS*, is mainly expressed in the quiescent centre and plays a role in maintaining stem cells of root apical meristem (RAM) [9]. *AtWOX6 *regulates ovule development [10], and *AtWOX9 *maintains cell division and inhibits SAM differentiation [11]. In rice (*Oryza sativa*), *OsWOX5* is involved in the specification and maintenance of the RAM stem cells and its expression is specifically detectable in the quiescent center of the root [12]. *OsWOX11* is expressed in emerging crown roots and later in cell division regions of the root meristem and is involved in the activation of crown root emergence and growth [13].

In *Arabidopsis*, 15 *WOX *genes are grouped into 3 clades: group 1 containing *WOX1*–*WOX7* and *WUS*; group 2 containing *WOX8*, *WOX9*, *WOX11*, and *WOX12*; group 3 containing *WOX10*, *WOX13*, and *WOX14 *[7, 14]. *WOX*s from monocots including rice, maize (*Zea mays*), *Brachypodium* (*Brachypodium distachyon*), and *Sorghum* (*Sorghum bicolor*) are divided into 3–5 clades [15–17]. Nardmann et al. (2009) analyzed the evolution of WOX family in two basal angiosperms (*Amborella trichopoda* and *Nymphaea jamesoniana*) and three gymnosperms (*Pinus sylvestris*, *Ginkgo biloba*, and *Gnetum gnemon*) and observed common ancestors of WOX members in monocots and dicots [18]. Deveaux et al. (2008) proposed that WOX13 subgroup probably represents the oldest clade among WOX families [19]. More recently, we divided WOX members of *Arabidopsis*, poplar (*Populus trichocarpa*), rice, maize, and *Sorghum* into nine subgroups of three clades and revealed multiplication/duplication of WOX families in these five plants during evolutionary process [14]. However, the origins and evolutionary history of WOX family in plant kingdom remain unclear.

In this study, we systematically performed the analysis of the origin and evolutionary history of 350 WOX sequences from 50 plants species. In addition, the conserved and the possible active amino acid residues located in the homeodomain were revealed. Moreover, we analyzed the expression and methylation of *WOX* genes in *Arabidopsis* and rice and discussed their possible roles.

## 2. Results

### 2.1. Identification of WOX Sequences in Plants

**Figure 1:**
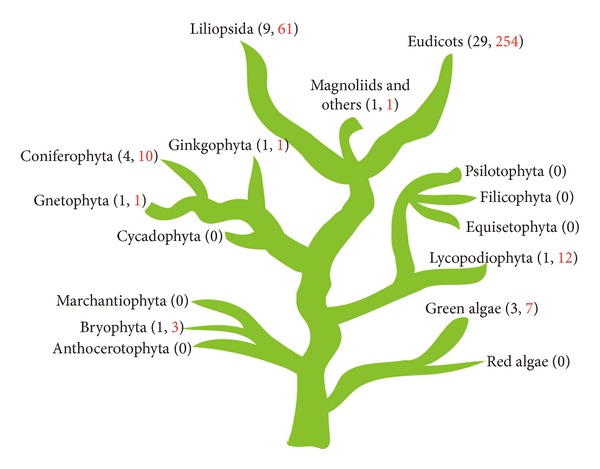
Summary of investigated plant species and their phylogenetic relationship. There were 350 WOX sequences of 50 species in 21 families from green algae to angiosperms. The numbers in black mean the amount of species and the numbers in red mean the amount protein sequences of WOX family. The species include *Arabidopsis lyrata*, *Arabidopsis thaliana*, *Arachis hypogaea*, *Artemisia annua*, *Brassica napus*, *Brassica rapa*, *Carica papaya*, *Citrus sinensis*, *Cucumis sativus*, *Glycine max*, *Gossypium hirsutum*, *Helianthus annuus*, *Lotus japonicus*, *Malus x domestica*, *Manihot esculenta*, *Medicago truncatula*, *Mimulus guttatus*, *Nicotiana tabacun*, *Petunia x hybrida*, *Phaseolus coccineus*, *Populus trichocarpa*, *Prunus persica*, *Raphanus sativus*, *Ricinus communis*, *Solanum lycopersicum*, *Solanum tuberosum*, *Theobroma cacao*, *Vigna unguiculata*, *Vitis vinifera*, *Brachypodium distachyon*, *Hordeum vulgare*, *Lycoris longituba*, *Oryza sativa*, *Panicum virgatum*, *Saccharum officinarum*, *Sorghum bicolor*, *Triticum aestivum*, *Zea mays*, *Amborella trichopoda*, *Ginkgo biloba*, *Gnetum gnemon*, *Picea abies*, *Picea sitchensis*, *Pinus sylvestris*, *Pinus taeda*, *Selaginella moellendorffii*, *Physcomitrella patens*, *Micromonas*, *Ostreococcus tauri*, and* Ostreococcus lucimarinus*.

To obtain sequences of WOX family in plant kingdom, we used the full-length sequence of *AtWUS* (*At2g17950)* [7] as a TBLASTN query against the available genome sequences (see Supplementary Table 1 in Supplementary Materials available online at http://dx.doi.org/10.1155/2013/534140) including the unicellular green algae (3 species), the bryophyte (1 species), the Lycopodiophyta (1 species), the Gnetophyta (1 species), the coniferophyta (4 species), the Ginkgophyta (1 species), and flowering plants (39 species). Generally, candidate sequences containing the characteristic homeodomain with higher similarity were identified. Further, the homologous and conserved sequences were manually reconstructed by repeated sequence alignment. A total of 367 putative WOX family proteins with *E* values below <1*e* − 5 were identified from the databases of TAIR (The *Arabidopsis* Information Resource database), RGAP (the Rice Genome Annotation Project database), JGI (genomic databases in Joint Genome Institute Eukaryotic Genomics), and PlantTFDB (the Plant Transcription Factor Database). Redundant members were removed according to Sol Genomics Network (SGN) (http://solgenomics.net/), UniProt (http://www.uniprot.org/), SMART (http://smart.embl-heidelberg.de/), and Pfam (http://pfam.sanger.ac.uk/), and eventually 350 WOX proteins from 50 species in 21 families ranging from green algae to angiosperms were obtained, that is, 3 species (*Micromonas pusilla*, *Ostreococcus tauri*, and *Ostreococcus lucimarinus*) in Chlorophyta, 1 species (*Physcomitrella patens*) in Bryophyta, 1 species (*Selaginella moellendorffii*) in Lycopodiophyta, 6 species in gymnosperms, 29 species in eudicots, and 9 species in monocots (Figure 1 and Supplementary Table 1). 244 of the WOX sequences have not been defined in previous publications, for example, WOX15 (AT5g46010) from *Arabidopsis*. Interestingly, we observed that several different genes encode the same WOX proteins; that is, *LOC_Os11g01130* and *LOC_Os12g01120* encode rice OsWOX3 (Supplementary Table 1). Additionally, the lower plants such as green algae and moss have fewer *WOX* genes than those of higher plants: 2 members found in *Ostreococcus*, 3 members in *Physcomitrella patens*, and above ten members in most of the higher plants. This suggested that the WOX members expanded as the evolution of plants.

Previously, various names were used in WOXs [8, 14–16, 18–21]; we used the WOX names described by Zhang et al. (2010) [14] and the LOCUS number (RGAP, http://rice.plantbiology.msu.edu/; JGI, http://genome.jgi-psf.org/) to avoid confusion.

### 2.2. Multiple Sequences Alignment and Analysis of Conserved Residues/Domains

**Figure 2:**
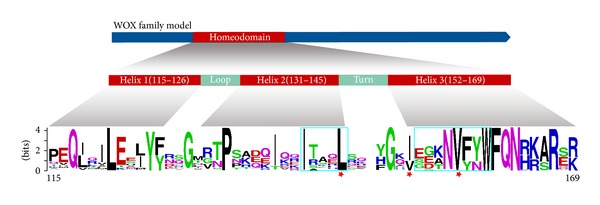
Alignment of the WOX homeodomain sequences. Canonical conserved residues analyzed by WebLogo (Web-based sequence logo generating application; Weblogo.berkeley.edu). The homeodomain of WOX family contained the helix-loop-helix-turn-helix structure [12]. The highly conserved residues are revealed by the alignment of homeodomains, the three highest conserved residues were marked using asterisk, and the residues within the two boxed motifs within the homeodomain marked using azure have close positional correlation which is revealed in Figure 3.

To examine sequence features of these WOX family proteins, we conducted multiple sequence alignment of 350 WOXs (Figure 2 and Supplementary Figure 1). Generally, these 350 WOXs have the conserved amino acids among the homeodomain, and the average size of the homeodomain is 60 aa, and all the homeodomains contain the helix-loop-helix-turn-helix structure [12]. Previously, it has been reported that the homeodomain of WOXs contains 17 conserved amino acids [1, 14]. In this study, we observed 9 additional conserved residues, including E (122) and F (126) in helix 1; G (129) in loop; T (132), I (138), and T (142) in helix 2; N (156), Y (159), and A (166) in helix 3, among these WOX members (Figure 2).

**Figure 3:**
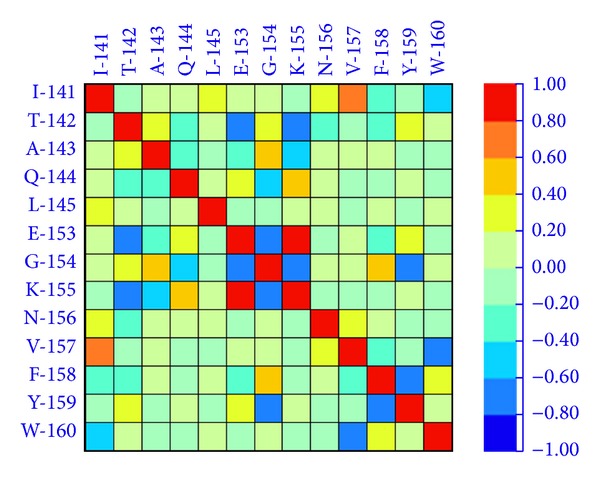
Pairwise positional correlation estimation of WOX sequences. The pairwise positional correlation estimation was analyzed by CRASP (http://www.bionet.nsc.ru/en). Two regions within the homeodomain, one is from 141 (I) to 145 (L) and the other is from 153 (E) to 160 (W), were closely correlated. The color refers correlation coefficiency of the amino acid at a certain position.

To understand the possible relationship between amino acid residues of WOXs and the function during the evolutionary change, the physicochemical value of amino acid sites was calculated using the modified version of an algorithm CRASP [22]. Physicochemical value reflects a significant correlation between the protein sequence and function possibly because of structural and functional constraints or results from evolutionary history and stochastic events [22]. The physicochemical analysis results showed that the pairwise positions from 141 (I) to 145 (L) correlated with the pairwise positions from 153 (E) to 160 (W) within the homeodomain in WOX family (Figure 3 and Supplementary Figure 2). In particular, the positive correlation coefficient between 153 (E) and 155 (K) was 0.813, and the positive correlation coefficient between 141 (I) and 157 (V) was 0.743, and there was a negative correlation between 158 (Y) and 159 (N) with a correlation coefficient of 0.770. This result suggested that these amino acids may be required for the function of the homeodomain during the evolutionary change.

### 2.3. Phylogenetic Analysis of WOXs

**Figure 4:**
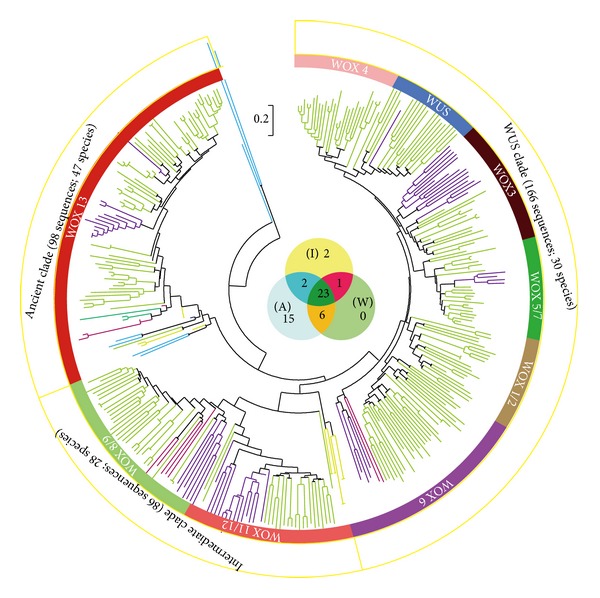
Phylogenetic analysis of WOX family. A simplified version of the neighbor joining (NJ) tree, with 350 sequences of proteins from 50 species from green algae to angiosperms. The tree was divided into three clades. The three clades were the WUS clade, containing 166 sequences, 30 species; the intermediate clade, containing 86 sequences, 28 species; the ancient clade, containing 98 sequences, 47 species. The full phylogeny is shown in Supplementary Figure 3. In the inset, (A), (I), and (W) refer to the ancient clade, the intermediate clade, and the WUS clade. Two species (*P*. *staeda* and *S*. *moellendorffii*) have members in both ancient clade and intermediate clade; one species (*P*. *hybieda*) has members in both intermediate clade and WUS clade; six species (*B*. *rapa*, *Gossypium hirsutum*, *P*. *sitchensis*, *R*. *sativus*, *Solanum tuberosum*, and *Vigna unguiculata*) have members in both ancient clade and WUS clade; twenty-three species have members belonging to three clades. The species names are shown in Supplementary Table 2.

To understand the evolutionary change of WOXs, we conducted phylogenetic analyses using the full-length sequences of all 350 sequences. Although some bootstrap values for interior branches were low because of the large number of sequences included [23], a relatively well-supported phylogenetic tree was obtained (Figure 4 and Supplementary Figure 3). The phylogenetic tree constructed using the full length of WOX sequences was nearly identical to that by the WOX homeodomain, and we thus only show the phylogenetic tree conducted using the full-length sequences (Figure 4 and Supplementary Figure 3). These 350 WOX members were divided into three clades, and the first clade (also called the ancient clade) contained 98 WOXs in 47 species from lower plants to seed plants, including 7 WOXs from green algae, 3 WOXs from bryophyta, 6 WOXs from Lycopodiophyta, 1 WOX from Gnetophyta, 3 WOXs from coniferophyta, 1 WOX from Ginkgophyta, 1 WOX from *Amborella trichopoda*, 13 WOXs from Liliopsida, and 63 WOXs from eudicots, which are homologous to *Arabidopsis *WOX10, WOX13, and WOX14.

The second clade (also called the intermediate clade) consisted of 86 WOXs homologous to *Arabidopsis* WOX8, WOX9, WOX11, and WOX12, which are only from 28 vascular plant species, that is, 6 from Lycopodiophyta, 5 from coniferophyta, 23 from Liliopsida, and 52 WOXs from eudicots. These members in the intermediate clade were further divided into two subgroups, designed WOX8/9 and WOX11/12. WOX8/9 contained 42 members and WOX11/12 with 44 members. The third clade (also called the WUS Clade) contained 166 WOXs including *Arabidopsis* WUS which are only from 30 seed plants. These 166 members had 2 WOXs from coniferophyta, 25 WOXs from Liliopsida, and 139 WOXs from eudicots.

### 2.4. Motifs of WOXs

**Figure 5:**
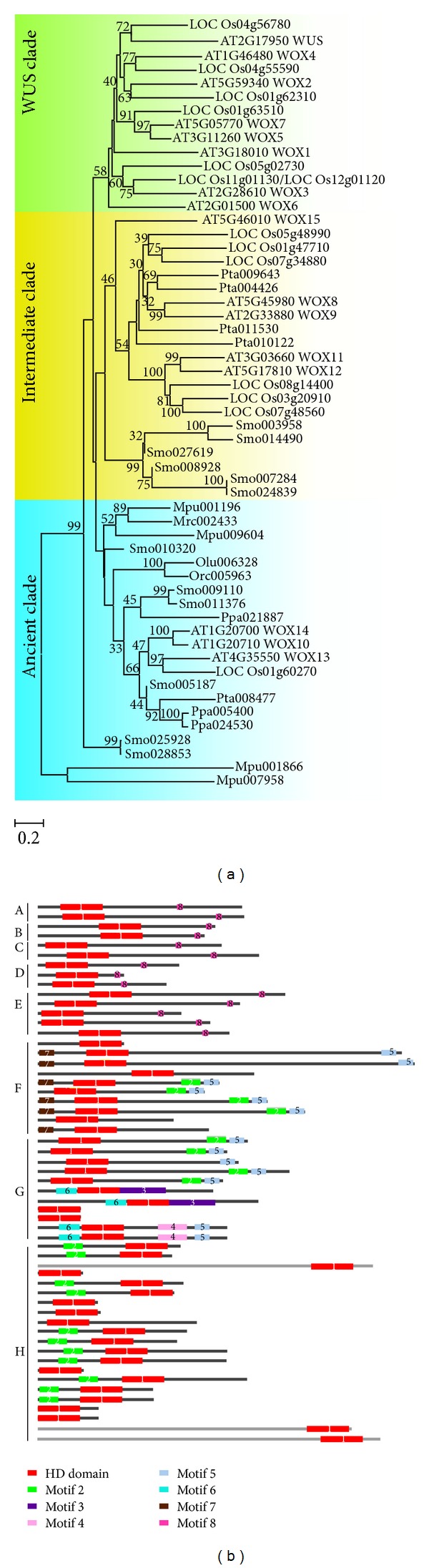
Phylogenetic tree of WOX family and motif analysis in model plants. (a) The phylogenetic tree (NJ) was constructed by MAGE 4 (Kumar et al., 2004) using 56 sequences from green algae (*Micromonas*, *Ostreococcus tauri*, *and Ostreococcus lucimarinus*), *Physcomitrella patens *(Ppa), *Selaginella moellendorffii* (Smo), *Pinus taeda* (Pta), *Oryza sativa* (Os), and *Arabidopsis thaliana* (At). WUS clade, intermediate clade, and ancient clade are color-coded green, orange, and blue, respectively. The WUS clade contains members only from angiosperms; the intermediate clade contains members only from vascular plants; the ancient clade contains members from lower plants to higher plants. (b) The conserved motifs among the members are highlighted in colored boxes with an arranged number and the sequences of the motifs are listed in Supplementary Table 3.

To further understand how WOX family evolved in plants and the protein sequence change, we selected 56 WOX members from model plants including *Arabidopsis*, rice, loblolly (*Pinus taeda*),* S*. *moellendorffiii*, moss (*P. patens*), *Ostreococcus*, and *Micromonas* for phylogenetic analysis (Figure 5(a)). Consistently, these WOX members were also divided into three clades: the ancient clade, the intermediate clade, and the WUS clade. The ancient clade contained the members from green algae, moss, and vascular plants; the intermediate clade contained members from vascular plants; the WUS clade only contained the members from seed plants, confirming the evolutionary relationship of WOXs.

Furthermore, we observed that most of the members in the same clade shared one or more common motifs besides the homeodomain. The multiple EM for motif elicitation tool (MEME, http://meme.nbcr.net/meme/) and Surveyed Conserved Motif Alignment Diagram and the Associating Dendrogram Database (SALAD database, http://salad.dna.affrc.go.jp/salad/en/) were used to identify the similar motifs among WOXs. In addition to the homeodomain, a total of 7 motifs were observed in these members from the 56 WOX members (Figure 5(b) and Supplementary Table 3), and most of these motifs have not yet been characterized. The proteins in the same cluster display the same or similar domain organization, suggesting the reliable phylogenetic analysis [24]. In the ancient clade, three WOX members from green algae, one WOX member from moss, and all WOXs members from *S*. *moellendorffii* were observed to only have one domain, that is, the homeodomain, suggesting that they may represent the ancestral form of WOXs. Furthermore, other members of ancient clade from green algae, moss, loblolly, *Arabidopsis*, and rice were observed to contain another motif (number 2 motif) at the N-terminus of the WOX sequences, indicating that these WOXs might gain additional motifs after the divergence from the ancestor. Compared with the ancient clade, the motif distribution of members in the intermediate clade seemed more diversified and seven motifs were observed, that is, except the homeodomain, two motifs were located at the N-terminus of the WOX sequences while four were at the C-terminus of the WOX sequences (Figure 5(b)). No. 2 motif located at the N-terminus of the WOX sequences in the ancient clade was also observed at the C-terminus in eight members of seed plants (loblolly, *Arabidopsis*, and rice), suggesting the conserved role of this motif during the evolution. Interestingly, No. 5 motif is close to No. 2 motif among the intermediate clade members except that three rice members (LOC_Os01g47710, LOC_Os05g48990, and LOC_Os07g34880) which didn't have No. 2 motif. Furthermore, two members (Smo008928 and Smo027619) of WOX family from *S. moellendorffii *only had the homeodomain; however, the other numbers in *S. moellendorffii *had more than one motif. In seed plants, six members of the intermediate clade had an extra new motif (No. 7 motif) at the N-terminus of the WOX sequences. All the members of WUS clade contained two motifs: the homeodomain and WUS box (No. 8 motif) (Figure 5(b)). This observation suggested that formation of motif is associated with the subfunctionalization and neofunctionalization of WOX members.

**Figure 6:**
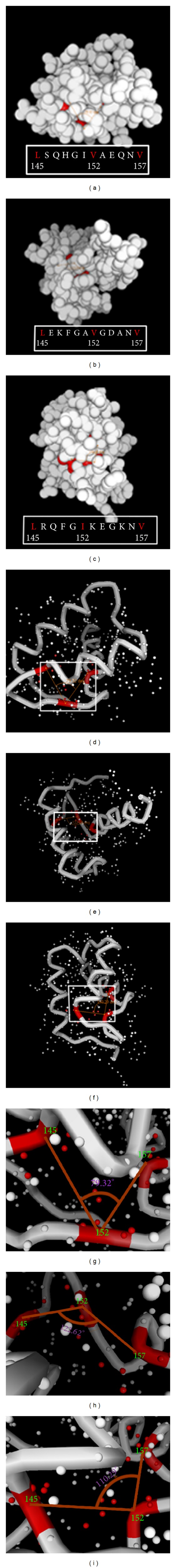
3D structures of the homeodomain in different clades of WOXs. All the WOX proteins had 3 conserved residues in the homeodomain: 145 (L), 152 (V/I), and 157 (V). The 3 residues formed an angle in (a), (d), and (g) 3D structures of ancient clade proteins (WOX13): (b), (e), and (h) 3D structures of intermediate clade proteins (WOX11); (c), (f), and (i) 3D structures of WUS clade proteins (WUS); 79.32 deg, 122.62 deg, and 110.29 deg, respectively.

To understand the relationship between WOX function and evolutionary events, we analyze the three highly conserved residues in the homeodomain: L (145), I (152), and V (157) (Figures 2 and 6). 3D-structure prediction of the homeodomain showed that these three amino acids were located in the interior of the homeodomain (Figure 6), implying that these three residues may perform key roles. Moreover, the angles in 3D structure formed by the three residues of ancient clade proteins (WOX13), intermediate clade proteins (WOX 11), and WUS clade proteins (WUS) were 79.32 degree, 122.62 degree, and 110.29 degree, respectively. The result suggested that the homeodomain 3D structures from different clades have differences even though they share similar primary structures. It is obvious that the angle in ancient clade was smaller than those of the intermediate clade and the WUS clade. It may be from the functional change of WOX family during the evolution.

### 2.5. Evolution of WOX Family

The observation that lower plants only have the WOX members from the ancient clade and that the members from the WUS clade were only observed in higher plants (Figures 4 and 5; Supplementary Figure 3 and Supplementary Table 2) suggested that the ancient clade represents the ancient WOX members, and the members in the intermediate clade and the WUS clade formed subsequently by gene duplication and diversification from the ancient members during the evolutionary history. Statistically, the average number of WOXs per species in the ancient clade, the intermediate clade, and the WUS clade is 2.09, 3.07, and 5.53, respectively.

The presence of the homeodomain of the WOX proteins from in extant eukaryotes from the algae to flowering plants supported the previous hypothesis that this DNA-binding domain might be originated before the divergence of the eukaryotes [25]. The phylogenetic analysis suggested that there was at least one WOX member as the last common ancestor among the green algae and land plants (Figures 4 and 5). To better understand how WOX family has evolved in plants, we analyzed the MRCA (most recent common ancestor) of *O. tauri*, *P. patens*, *S. moellendorffi*, *P. taeda*, *rice*, and* Arabidopsis* and deduced that WOX family originated from the ancient clade and the members in the ancient clade evolved independently among plant species. The WOX members of green algae, Bryophyta, Gnetophyta, Ginkgophyta, and *Amborella trichopoda* obtained by our query conditions were divided into the ancient clade, and members of intermediate clade and the WUS clade were not observed in nonvascular plants, confirming the ancient and conserved role of the ancient clade.

**Figure 7:**
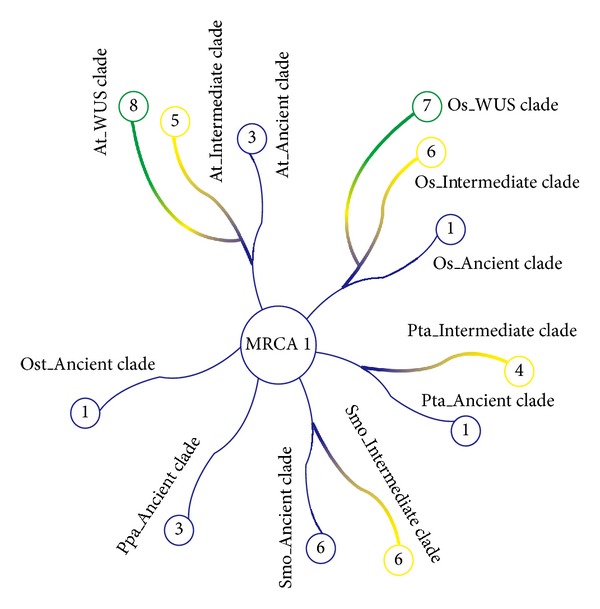
Evolutionary change of the number of WOX proteins in model plants. The numbers in circles represent the numbers of genes in extant species. Ost: *Ostreococcus tauri*, Ppa: *Physcomitrella patens*, Smo: *Selaginella moellendorffii*, Pta: *Pinus taeda*, Os: *rice*, and At: *Arabidopsis thaliana*.

The phylogenetic tree showed that the first expansion of members in the intermediate clade from the ancient clade ancestor occurred in plants from ferns to higher plants (Figure 7). Subsequently, the ancestor of intermediate clade might have undergone a duplication and formed two subgroups: WOX8/9 and WOX11/12 in vascular plants (Figures 4, 5, and 7). Furthermore, all WOX members in the WUS clade except the subgroup WOX6 containing WOX members from coniferophyta were only observed in flowering plants, suggesting that the WUS clade plays a key role during the evolution of higher plants, and the WOX6 subgroup may represent the oldest subgroup in the WUS clade. Additionally, the WOX1/2 subgroup appeared to be generated from the WOX6 subgroup, and the WOX5/7 subgroup might be originated from the WOX1/2 subgroup.

### 2.6. Expression Analyses of *WOXs *in *Arabidopsis *and Rice and the Predictive DNA Methylated Region

**Figure 8:**
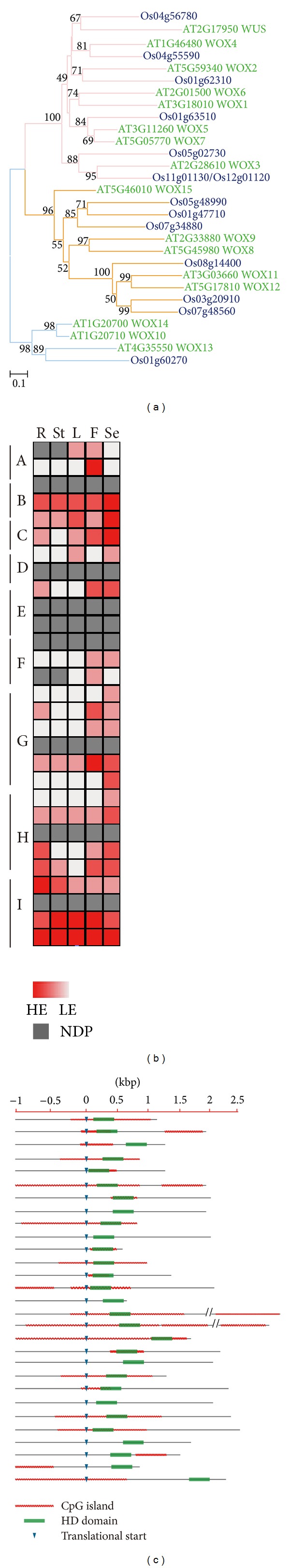
Phylogenetic and expression analysis of WOX family in* Arabidopsis* and rice. (a) The phylogenetic tree (NJ) of 29 WOX members from *Arabidopsis* and rice constructed by MAGE 4 (Kumar et al., 2004). A, B, C, D, E, F, G, H, and I refer to subgroups WUS, WOX4, WOX2, WOX1/6, WOX5/7, WOX3, WOX8/9, WOX11/12, and WOX13, respectively. (b) Expression pattern analysis; the average values were chosen among the expression values published in AtGenExpress. (http://www.weigelworld.org/resources/microarray/AtGenExpress), SIGnAL (http://signal.salk.edu/), and RiceXPro (http://ricexpro.dna.affrc.go.jp). R: root; St: stem; L: leaf; F: flower; Se: seed; HE: high expression; LE: low expression; NDP: no data published. (c) The CpG islands among the members are highlighted in red lines.

To better understand the duplication event in *Arabidopsis* and rice, we constructed a phylogenetic tree of 29 WOXs from these two species (Figure 8(a)). The phylogenetic tree contained three clades: the ancient clade, the intermediate clade, and the WUS clade. We observed relatively low bootstrap values in interior branches of the WUS clade, which is consistent with previous reports [14, 19], suggesting that WUS clade has more diversified members. In the WOX3 subgroup of the WUS clade, there were three rice genes and one from *Arabidopsis*, and their phylogenetic role suggested that WOX3 subgroup duplicated before and after the divergence of rice, or the homolog(s) of LOC_Os05g02730 in *Arabidopsis *was (were) lost during evolution. In the WOX5/7 subgroup of the WUS clade, *Arabidopsis* contained two members and one in rice, suggesting that rice might lose one member during evolution or this subgroup in *Arabidopsis* duplicated recently. In the WOX1/6 subgroup of the WUS clade, no homologs were found in rice which is consistent with a previous study by Zhang et al. (2010), suggesting that WOX1/6 subgroup in rice was lost. The subgroups, WUS, WOX4, and WOX2, contain only one individual member with high bootstrap values from *Arabidopsis* and rice, respectively, implying that these members may play a conserved and crucial role. In the WOX11/12 subgroup of the intermediate clade, members of rice formed one branch and members of *Arabidopsis* formed another one, suggesting that both rice and *Arabidopsis* underwent one duplication after the divergence of them. In the ancient clade, three members of *Arabidopsis* and one member of rice were grouped into two separated branches with high bootstrap values. One branch contained AtWOX13 and LOC_Os1g60270, and the other contained AtWOX10 and AtWOX14, suggesting that AtWOX13 and LOC_Os1g60270 may play a conserved role, and AtWOX10 and AtWOX14 were generated by duplication.

To understand the function of WOXs, we investigated the expression pattern of *AtWOX*s and *OsWOX*s and the methylation information of their promoter regions using the available dataset from AtGenExpress (http://www.weigelworld.org/resources/microarray/AtGenExpress), RiceXPro (http://ricexpro.dna.affrc.go.jp), and SIGnAL (Salk Institute Genomic Analysis Laboratory; http://signal.salk.edu/) (Figure 8(b)). Consistent with previous observation [14], wide expression of *WOX* genes from *Arabidopsis *and rice was detectable in roots, stems, leaves, flowers, and seeds, suggesting that these WOXs play regulatory roles at various developmental events. Furthermore, some *WOX *homologs showed conserved expression pattern; for instance, members of the ancient clade *AtWOX13* and *AtWOX14 *and *OsWOX13* (*LOC_Os01g60270*) were highly expressed in different organs (Figure 8(b)). In the intermediate clade, *WOX8* and *WOX9 *exhibited detectable expression signals in seeds, and *WOX9 *also in flowers; *WOX15 *(*AT5g46010*), *OsWOX9c* (*LOC_Os05g48990*), and *OsWOX9a* (*LOC_Os01g47710*) had lower expression levels in various tissues except flower, where *OsWOX9c* (*LOC_Os05g48990*) was expressed higher than other tissues (Figure 8(b)). Moreover, the WUS clade members exhibited higher expression in flowers (Figure 8(b)), suggesting that these members play an important role in the development of flowers, consistently with previous observation of activation role of WUS in floral patterning. Furthermore, the expression of *OsWOX4 *(*LOC_Os4g55590*), *WOX2*, and *OsWOX2 *(*LOC_Os1g62310*) was highly detectable in seeds.

DNA methylation is closely associated with the transcriptional regulation of gene expression [26]. Recent studies showed that the expression of *WUS *is regulated by DNA methylation, and there are three characteristic epigenetic marks of DNA methylation, that is, CpG motif within the *WUS* genomic sequences [27]. The sequences of one-kilobase (kb) promoter fragment and the genomic DNA region of *Arabidopsis* and rice *WOXs* were analyzed and CpG islands were observed in promoter regions of 10 *Arabidopsis *WOXs and 18 CpG islands within the promoter regions of 13 rice *WOXs. *In addition, 7 CpG islands were seen in the homeodomain of *Arabidopsis WOXs*, and 12 in the homeodomain of rice *WOXs *(Figure 8(c)). This observation suggests that *WOXs* may share epigenetic methylation modification modulating their expression during evolution.

## 3. Discussions

### 3.1. WOXs May Originate in Green Algae

Evolution created a tremendous variation in organ shapes within the plant kingdom. Plant diverse morphologies are associated with the activity of stem cells, which are regulated by WOX genes such as WUS and WOX5 in model eudicot *Arabidopsis* for maintaining stem cell in the shoot and the root, respectively [28]. In this study, we revealed 244 previously undefined WOX sequences. Our phylogenetic analysis using the 350 WOXs family members from 50 plant species supports that WOX gene family has a monophyletic origin [8, 14–16, 18–21]. Previous evolutionary analyses of WOX family genes using limited available genome sequences [18, 19, 29, 30] proposed that the green alga WOX genes may represent the earliest WOXs. We collected WOX family sequences using 3 green algal species: *Micromonas pusilla*, *Ostreococcus lucimarinus*, and *Ostreococcus tauri*, and comprehensive analysis supports the notion that WOX proteins in green alga represent the oldest members in WOX family. Supportively, we did not find out any WOX family gene in the genome of *Cyanidioschyzon merolae*, which belongs to the red algae group and is supposed to be earlier than green alga during evolution, even though we can not exclude the possibility that the red alga species lost WOXs during the evolution.

**Figure 9:**
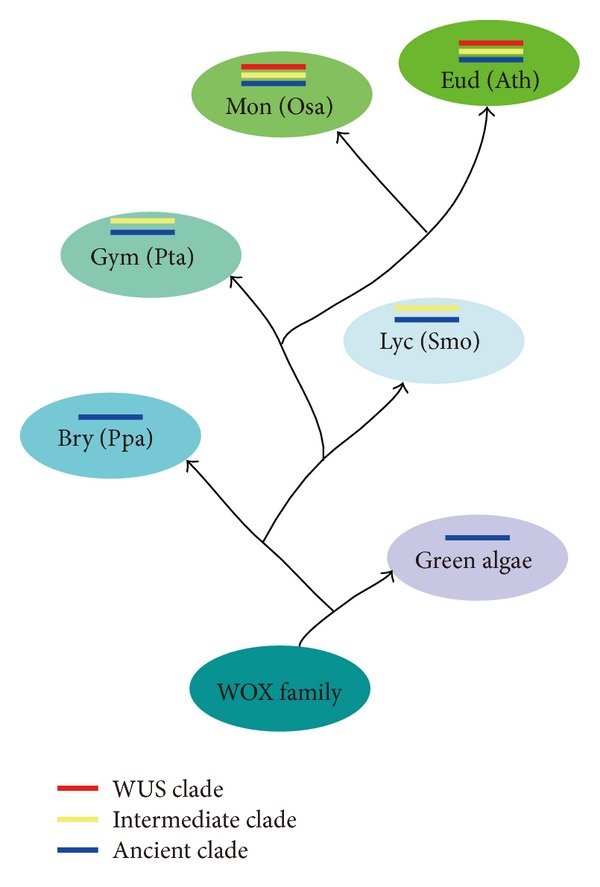
A proposed model of the evolutionary history of plant WOX family. The schematic tree represents the proposed evolutionary history of WOX family. Different color represents different clade. The WOX family might originate from green algae and expand in Lycopodiophyta with the appearance of the intermediate clade, and along with the emergence of seed plants, the WOX family generated WUS clade from the intermediate clade. Bry: Bryophyta, Lyc: Lycopodiophyta, Gym: Gymnosperm, Mon: Monocots, Eud: Eudicots, Ppa: *Physcomitrella patens*, Smo: *Selaginella moellendorffii*, Pta: *Pinus taeda*, Osa: *Oryza sativa*, and Ath: *Arabidopsis thaliana*.

In addition, our phylogenetic analysis revealed that the ancient clade is the most ancient one and the WUS clade represents the latest members, which is consistent with previous analysis of WOX family in* Arabidopsis *[7] as well as other phylogenetic analyses [8, 14, 19–21]. Consistently, the subclade encompassing WOX13 is considered the oldest one [19] and WUS/WOX5 as the modern one (Figure 9) [18].

### 3.2. The Homeodomain Region Plays a Key Role in Plant Development

The homeodomain can recognize sequence-specific targets in a precise spatial and temporal pattern, and helix 3 plays an important role in this process [1]. We did linkage analysis on WOX amino acid sequences in the plant kingdom and showed the correlation between amino acids in the homeodomain region, suggesting the importance of these residues to the role of the homeodomain in WOX family. Particularly, we observed that all the WOX proteins have three highly conserved residues in the homeodomain: L (145), I (152), and V (157), and the homeodomain 3D structures have differences in different clade, suggesting the reliability of the phylogenetic analysis. Moreover, we observed the putative methylated regions of the promoter and the homeodomain-encoding sequences, suggesting that the homeodomain may be modulated by epigenetic marks and contributing to the expression control of WOXs.

## 4. Methods

### 4.1. Search of WOX Members

WOX family proteins were retrieved by TBLASTN using the following databases: the National Center for Biotechnology Information (NCBI) database, The *Arabidopsis* Information Resource (TAIR) database, the Rice Genome Annotation Project (RGAP) database, genomic databases in Joint Genome Institute (JGI) Eukaryotic Genomics, and the Plant Transcription Factor Database (PlantTFDB). We used the full-length sequence of AtWUS as a query sequence for TBLASTN. The *E* value of all the sequences we obtained was below 1*e* − 5. The structure and function of all the sequences were also checked to remove the redundant and non-WOX family sequences using the Sol Genomics Network (SGN) database, the UniProt, the SMART, and Sanger database, respectively.

### 4.2. Multiple Sequence Alignments

Multiple sequence alignments were carried out by using MUSCLE 3.6 with the default parameter setting. In order to obtain a better alignment, we adjusted manually the results based on the location of the corresponding amino acids in the WOX motif using GeneDoc (version 2.6.002) software.

### 4.3. Construction of Phylogenetic Tree

A phylogenetic tree using neighbor joining method was constructed with the aligned WOX protein family sequences by MEGA (version 3.0;). NJ analyses were done using the following parameters: poisson correction methods, pairwise deletion of gaps, and bootstrap (1000 replicates; random seed).

### 4.4. Expression Analysis of AtWOXs and OsWOXs

Expression pattern data of AtWOXs and OsWOXs were obtained from the following databases: AtGenExpress Visualization Tool (AVT) and RiceXPro, respectively. The average values were calculated among the expression values of the organs.

### 4.5. Prediction of Methylated Region of AtWOXs and OsWOXs

Methyl Primer Express Software was used with the following parameter setting: minimum length of island is 300 bp, C + Gs/Total bases >50%, and CpG observed/CpG expected >0.6.

### 4.6. Analysis of Pairwise Positional Correlations

Analysis of pairwise positional correlations was obtained from the Correlation Analysis of Protein Sequences (CRASP) database with the default parameter setting.

## Supplementary Material

Supplementary Table 1: Plant genomes used for the analysis of WOXs in this study.
Supplementary Table 2: Each clade contains the numbers of species.
Supplementary Table 3: Sequences of each motif in WOX from model plants including green algae, *Physcomitrella patens, Selaginella moellendorffii, Pinus taeda*, rice and *Arabidopsis thaliana*.
Supplementary Figure 1: Alignment of full-length sequences of 350 WOXs.
Supplementary Figure 2: Pairwise positional correlation estimation of WOX proteins.
Supplementary Figure 3: The full phylogeny of WOX family.
