# Supplementary material for: Origins and Evolution of WUSCHEL-Related Homeobox Protein Family in Plant Kingdom
Source: ScientificWorldJournal. 2014 Jan 6;2014:534140. doi: 10.1155/2014/534140 (PMC3913392; doi:10.1155/2014/534140)
Supplement: Supplementary file 1 — Supplementary Table 1: Plant genomes used for the analysis of WOXs in this study. Supplementary Table 2: Each clade contains the numbers of species. Supplementary Table 3: Sequences of each motif in WOX from model plants including green algae, Physcomitrella patens, Selaginella moellendorffii, Pinus taeda, rice and Arabidopsis thaliana. Supplementary Figure 1: Alignment of full-length sequences of 350 WOXs. Supplementary Figure 2: Pairwise positional correlation estimation of WOX proteins. Supplementary Figure 3: The full phylogeny of WOX family. [file 534140.f1.docx]

**Supplementary Material**

| Kingdom | | Species | Name | Reference |
| --- | --- | --- | --- | --- |
| Angiosperm | Eudicots | *Arabidopsis lyrata* | Aly001008  Aly002688  Aly005751  Aly007078  Aly013629  Aly014809  Aly015918  Aly016712  Aly017355  Aly018117  Aly027052  Aly028161  Aly029745  Aly032086 | This study |
|  |  | *Arabidopsis thaliana* | AT1G20700  AT1G20710  AT1G46480  AT2G01500  AT2G17950  AT2G28610  AT2G33880  AT3G03660  AT3G11260  AT3G18010  AT4G35550  AT5G05770  AT5G17810  AT5G45980  AT5G46010  AT5G59340 | Haecker et al., 2004; This study |
|  |  | *Arachis hypogaea* | Ahy002590 | This study |
|  |  | *Artemisia annua* | Aan003821 | This study |
|  |  | *Brassica napus* | Bna017374  Bna007789  Bna020170  Bna010801  Bna006212  Bna018146  Bna002896 | Deveaux et al., 2008; This study |
|  |  | *Brassica rapa* | Bra005748  Bra004033  Bra001224 | Deveaux et al., 2008; This study |
|  |  | *Carica papaya* | Cpa003844  Cpa003999  Cpa005434  Cpa005810  Cpa007655  Cpa011514  Cpa017776  Cpa018093  Cpa019809  Cpa023809  Cpa024891 | This study |
|  |  | *Citrus sinensis* | Csi006374  Csi001922  Csi011569  Csi006573  Csi002594  Csi009198 | Deveaux et al., 2008; This study |
|  |  | *Cucumis sativus* | Csg002899  Csg003968  Csg005213  Csg005879  Csg009511  Csg011313  Csg011479  Csg012885  Csg015032  Csg015381  Csg015592  Csg017854  Csg026558 | This study |
|  |  | *Glycine max* | Glyma01g37190  Glyma02g10410  Glyma02g42200  Glyma03g01000  Glyma04g01830  Glyma04g04310  Glyma05g33850  Glyma06g01940  Glyma06g04470  Glyma07g11370  Glyma07g15710  Glyma07g32430  Glyma07g34420  Glyma08g05830  Glyma09g30830  Glyma10g08030  Glyma10g43580  Glyma11g08090  Glyma11g14940  Glyma11g34990  Glyma12g06890  Glyma13g21860  Glyma13g24150  Glyma13g41000  Glyma14g09310  Glyma15g04460  Glyma17g35880  Glyma18g03350  Glyma18g39520  Glyma18g52490  Glyma19g29660  Glyma20g02160  Glyma20g23220 | Deveaux et al., 2008; This study |
|  |  | *Gossypium hirsutum* | Ghi017496  Ghi006586  Ghi016256  Ghi006162  Ghi011650  Ghi006185 | Deveaux et al., 2008; This study |
|  |  | *Helianthus annuus* | Han002587 | Deveaux et al., 2008 |
|  |  | *Lotus japonicus* | Lja004339  Lja007138  Lja007565  Lja007659  Lja014335  Lja014933  Lja016202  Lja017235  Lja017305  Lja023662  Lja024882  Lja026877  Lja026884 | Deveaux et al., 2008; This study |
|  |  | *Malus x domestica* | Mdo013211  Mdo008276 | Deveaux et al., 2008; This study |
|  |  | *Manihot esculenta* | Mes003838  Mes004202  Mes005210  Mes009836  Mes018961  Mes018967  Mes025377  Mes026108  Mes026651  Mes026976  Mes028632  Mes031191  Mes033638  Mes035354  Mes036319  Mes037898  Mes042700  Mes043505  Mes044258  Mes045144 | This study |
|  |  | *Medicago truncatula* | Medtr1g024140  Medtr2g018480  Medtr4g144750  Medtr4g145020  Medtr4g154980  Medtr5g021830  Medtr5g089330  Medtr7g025190  Medtr7g026130  Medtr7g067850  Medtr7g103730 | Deveaux et al., 2008; This study |
|  |  | *Mimulu sguttatus* | Mgu003406  Mgu004235  Mgu004809  Mgu005008  Mgu005224  Mgu007211  Mgu007817  Mgu008424  Mgu009854  Mgu011052  Mgu013004  Mgu023740  Mgu025684 | This study |
|  |  | *Nicotiana tabacun* | Nta013065 | This study |
|  |  | *Petunia x hybieda* | ABO93066  ABO93067  ACA64093 | This study |
|  |  | *Phaseolus coccineus* | ACL11801 | This study |
|  |  | *Populus trichocarpa* | POPTR_0011s05980  POPTR_0004s05090  POPTR_0019s05730  POPTR_0013s06310  POPTR_0015s04520  POPTR_0012s04510  POPTR_0010s19950  POPTR_0005s27410  POPTR_0014s02540  POPTR_0002s12560  POPTR_0009s03460  POPTR_0008s06560  POPTR_0001s24470  POPTR_0007s14130  POPTR_0010s12150  POPTR_0005s11680 | Zhang et al., 2010;  This study |
|  |  | *Prunus persica* | Ppe001631  Ppe002043  Ppe004001  Ppe005188  Ppe010670  Ppe012457  Ppe014557  Ppe018604  Ppe025193  Ppe026296 | This study |
|  |  | *Raphanus sativus* | Rsa002287  Rsa000316  Rsa006600  Rsa007058  Rsa006061 | This study |
|  |  | *Ricinus communis* | Rco27471.t000009  Rco28102.t000001  Rco29669.t000014  Rco29816.t000028  Rco29830.t000069  Rco29876.t000002  Rco29900.t000017  Rco30039.t000016  Rco30072.t000001  Rco30170.t000258  Rco30170.t000801 | This study |
|  |  | *Solanum lycopersicum* | Sly000779  Sly012515  Sly011548  Sly008506  Sly006834  ACI23249  ACI23250 | This study |
|  |  | *Solanum tuberosum* | Stu005038  Stu004033 | Deveaux et al., 2008; This study |
|  |  | *Theobroma cacao* | Tca001557 | This study |
|  |  | *Vigna unguiculata* | Vun003771  Vun003357  Vun003266  Vun004930  Vun011016 | This study |
|  |  | *Vitis vinifera* | Vvi005050  Vvi010049  Vvi011406  Vvi011535  Vvi018586  Vvi022239  Vvi022263  Vvi024132  Vvi027636  Vvi030902  Vvi031384  Vvi031710  Vvi039921  Vvi040701  Vvi041451  Vvi041910  Vvi042690  Vvi044308  Vvi045340  Vvi045475  Vvi045702 | Deveaux et al., 2008; This study |
|  | Monocots | *Brachypodium distachyon* | Bdi002260  Bdi002407  Bdi002646  Bdi008512  Bdi012336  Bdi016474  Bdi017546  Bdi018003  Bdi018441 | Nardmann et al., 2007 |
|  |  | *Hordeum vulgare* | Hvu020129  Hvu023127  Hvu014144 | Deveaux et al., 2008; This study |
|  |  | *Lycoris longituba* | ADG57873 | This study |
|  |  | *Oryza sativa* | LOC_Os05g48990  LOC_Os01g47710  LOC_Os01g60270  LOC_Os01g62310  LOC_Os01g63510  LOC_Os03g20910  LOC_Os04g55590  LOC_Os04g56780  LOC_Os05g02730  LOC_Os07g34880  LOC_Os07g48560  LOC_Os08g14400  LOC_Os11g01130  LOC_Os12g01120 | Zhang et al., 2010 |
|  |  | *Panicum virgatum* | Pvi017728  Pvi008301  Pvi008059  Pvi007746 | This study |
|  |  | *Sacchayum officinarum* | Sof004312 | This study |
|  |  | *Sorghum bicolor* | Sb01g036670  Sb02g043300  Sb03g038080  Sb03g039380  Sb03g040210  Sb05g003490  Sb06g030700  Sb06g031880  Sb07g007600  Sb09g002010  Sb09g028562 | Zhang et al., 2010 |
|  |  | *Triticum aestivum* | Tae001397  Tae005051  Tae013429 | This study |
|  |  | *Zea mays* | AC155434  AC218972  GRMZM2G028622  GRMZM2G031882  GRMZM2G038252  GRMZM2G047448  GRMZM2G069028  GRMZM2G069274  GRMZM2G108933  GRMZM2G116063  GRMZM2G122537  GRMZM2G133972  GRMZM2G162481  GRMZM2G170958  GRMZM2G314064  GRMZM2G409881 | Nardmann et al., 2006; This study |
|  | Others | *Amborella trichopoda* | CAT02918 | Nardmann et al., 2009 |
| Gymnosperm | | *Ginkgo biloba* | CAT02909 | Nardmann et al., 2009 |
|  |  | *Gentum gnemon* | CAT02930 | Nardmann et al., 2009 |
|  |  | *Picea abies* | ADF42580 | This study |
|  |  | *Picea sitchensis* | Psi006453  Psi002276  Psi010097 | Deveaux et al., 2008; This study |
|  |  | *Pinu ssylvestris* | CAT02934 | Nardmann et al., 2009 |
|  |  | *Pinus taeda* | Pta009643  Pta004426  Pta008477  Pta011530  Pta010122 | Deveaux et al., 2008; This study |
| Lycopodiophyta | | *Selaginella moellendorffii* | Smo003958  Smo005187  Smo007284  Smo008928  Smo009110  Smo010320  Smo011376  Smo014490  Smo024839  Smo025928  Smo027619  Smo028853 | Nardmann et al., 2009; This study |
| Bryophyta | | *Physcomitrella patens* | Ppa005400  Ppa021887  Ppa024530 | Deveaux et al., 2008 |
| Green algae | | *Micromonas* | Mpu001196  Mpu001866  Mpu007958  Mpu009604  Mrc002433 | This study |
|  |  | *Ostreococcus tauri* | Olu006328 | Ikeda et al., 2009 |
|  |  | *Ostreococcus lucimarinus* | Orc005963 |  |

**Supplementary Table 1: Plant genomes used for the analysis of WOXs in this study.**

There were 350 protein sequences of 50 species in 21 families from green algae to angiosperms, and the identified sequences by previous studies were marked by underline.

| WUS Clade | Intermediate Clade | Ancient Clade |
| --- | --- | --- |
| ***Arabidopsis lyrata^1,2,3^***  *(Aly001008; Aly005751; Aly007078; Aly013629; Aly017355; Aly018117; Aly032086);*  ***Arabidopsis thaliana^1,2,3^***  *(AT1G46480; AT2G01500; AT2G17950; AT2G28610; AT3G11260; AT3G18010; AT5G05770; AT5G59340);*  ***Picea sitchensis^1,3^***  *(Psi006453; Psi010097);*  ***Sorghum bicolor^1,2,3^***  *(Sb03g039380; Sb03g040210; Sb05g003490; Sb06g030700; Sb06g031880; Sb09g002010);*  ***Triticum aestivum^1,2,3^***  *(Tae005051);*  ***Zea mays^1,2,3^***  *(GRMZM2G028622;GRMZM2G047448; GRMZM2G069028; GRMZM2G108933; GRMZM2G116063; GRMZM2G122537);*  ***Panicum virgatum^1,2,3^***  *(Pvi017728);*  ***Oryza sativa^1,2,3^***  *(LOC Os01g62310; LOC Os01g63510; LOC Os04g55590; LOC Os04g56780; LOC Os05g02730; LOC Os11g01130);*  ***Brachypodium distachyon^1,2,3^***  *(Bdi002260; Bdi017546; Bdi018003; Bdi018441);*  ***Hordeum vulgare^1,2,3^***  *(*Hvu020129*);*  ***Vigna unguiculata^1,3^***  *(Vun003771);*  ***Vitis vinifera^1,2,3^***  *(Vvi005050; Vvi011406; Vvi018586; Vvi024132; Vvi027636; Vvi031384; Vvi031710; Vvi040701; Vvi041451; Vvi041910; Vvi042690);*  ***Solanum tuberosum^1,3^***  *(Stu005038);*  ***Solanum lycopersicum^1,2,3^***  *(Sly000779; Sly008506; Sly012515);*  ***Populus trichocarpa^1,2,3^***  *(POPTR 0001s24470;*  *POPTR 0002s12560;*  *POPTR 0005s11680;*  *POPTR 0007s14130;*  *POPTR 0008s06560;*  *POPTR 0009s03460;*  *POPTR 0010s12150;*  *POPTR 0010s19950;*  *POPTR 0012s04510;*  *POPTR 0014s02540;*  *POPTR 0015s04520);*  ***Prunus persica^1,2,3^***  *(Ppe002043; Ppe004001; Ppe005188;Ppe012457; Ppe025193; Ppe026296);*  ***Raphanus sativus^1,3^***  *(Rsa000316; Rsa002287; Rsa006600);*  ***Ricinus communis^1,2,3^***  *(Rco27471t000009; Rco28102t000001; Rco29669t000014; Rco29816t000028; Rco29876t000002; Rco30039t000016; Rco30170t000258);*  ***Petunia x hybieda^1,2^***  *(ACA64093);*  ***Manihot esculenta^1,2,3^***  (Mes003838; Mes004202; Mes009836; Mes025377; Mes026108; Mes026651; Mes026976; Mes031191; Mes033638; Mes035354; Mes036319; Mes037898; Mes043505)*;*  ***Medicago truncatula^1,2,3^***  *(Medtr1g024140 Medtr4g144750 Medtr4g145020 Medtr4g154980*  *Medtr5g021830 Medtr5g089330*  *Medtr7g067850);*  ***Mimulus guttatus^1,2,3^***  *(Mgu003406; Mgu004235; Mgu004809; Mgu005224; Mgu007817; Mgu009854; Mgu011052; Mgu013004; Mgu025684);*  ***Lotus japonicas^1,2,3^***  *(Lja004339; Lja007138; Lja007565; Lja007659; Lja017305; Lja024882; Lja026884);*  ***Brassica napus^1,2,3^***  *(Bna007789; Bna017374; Bna020170);*  ***Brassica rapa^1,3^***  *(*Bra005748;*);*  ***Carica papaya^1,2,3^***  *(Cpa003999; Cpa005434; Cpa005810; Cpa011514; Cpa017776; Cpa023809; Cpa024891);*  ***Citrus sinensis^1,2,3^***  *(Csi006374);*  ***Cucumis sativus^1,2,3^***  *(Csg003968; Csg011313; Csg012885; Csg015032; Csg015381; Csg015592; Csg026558);*  ***Glycine max^1,2,3^***  *(Glyma01g37190; Glyma02g42200; Glyma05g33850; Glyma06g04470; Glyma07g11370; Glyma07g15710; Glyma07g34420; Glyma08g05830; Glyma09g30830; Glyma11g08090; Glyma11g14940; Glyma11g34990; Glyma12g06890; Glyma13g41000; Glyma14g09310; Glyma15g04460; Glyma17g35880; Glyma18g03350; Glyma18g39520; Glyma20g02160; Glyma04g04310);*  ***Gossypium hirsutum^1,3^***  *(Ghi006586; Ghi011650; Ghi016256;Ghi017496);* | ***Selaginella moellendorffii^2,3^***  *(Smo003958; Smo007284; Smo008928; Smo014490; Smo024839; Smo027619);*  ***Pinus taeda^2,3^***  *(Pta004426; Pta009643;*  *Pta010122; Pta011530);*  ***Picea abies^2^***  *(ADF42580);*  ***Sorghum bicolor^1,2,3^***  *(Sb01g036670*  *Sb02g043300*  *Sb07g007600*  *Sb09g028562);*  ***Triticum aestivum^1,2,3^***  *(Tae013429);*  ***Zea mays^1,2,3^***  *(GRMZM2G031882*  *GRMZM2G133972*  *GRMZM2G162481*  *GRMZM2G170958*  *GRMZM2G314064*  *GRMZM2G409881;AC1554342_FG007);*  ***Oryza sativa^1,2,3^***  *(LOC Os01g47710;*  *LOC Os03g20910;*  *LOC Os05g48990*  *LOC Os07g34880;*  *LOC Os07g48560;*  *LOC Os08g14400);* ***Panicum virgatum^1,2,3^***  *(Pvi008301);* ***Brachypodiumdistachyon^1,2,3^***  *(Bdi002407; Bdi008512; Bdi016474);*  ***Hordeum vulgare^1,2,3^***  *(Hvu023127);*  ***Vitis vinifera^1,2,3^***  *(Vvi011535;*  *Vvi022263;*  *Vvi030902; Vvi039921) ;*  ***Solanum lycopersicum^1,2,3^***  *(ACI23249; ACI23250; Sly011548);*  ***Ricinus communis^1,2,3^***  *(Rco29900t000017*  *Rco30072t000001);*  ***Populus trichocarpa^1,2,3^***  *(POPTR 0004s05090*  *POPTR 0011s05980*  *POPTR 0013s06310*  *POPTR 0019s05730);* ***Prunus persica^1,2,3^***  *(Ppe010670; Ppe014557);*  ***Petunia x hybieda^1,2^***  *(ABO93066; ABO93067);*  ***Phaseolus coccineus^2^***  *(ACL11801);*  ***Manihot esculent****a****^1,2,3^***  *(Mes005210; Mes018961; Mes042700; Mes045144);*  ***Medicagotruncatula^1,2,3^***  *(Medtr2g018480*  *Medtr7g026130*  *Medtr7g103730);* ***Mimulus guttatus^1,2,3^***  *(Mgu007211; Mgu008424; Mgu023740);*  ***Lotus japonicas^1,2,3^***  *(Lja016202; Lja023662);*  ***Carica papaya^1,2,3^***  *(Cpa007655; Cpa018093);*  ***Citrus sinensis^1,2,3^***  *(Csi001922);*  ***Cucumis sativus^1,2,3^***  *(Csg005879; Csg011479; Csg017854);*  ***Glycine max^1,2,3^***  *(Glyma03g01000*  *Glyma07g32430*  *Glyma10g08030*  *Glyma13g21860*  *Glyma13g24150 Glyma19g29660);*  ***Brassica napus^1,2,3^***  *(Bna010801);*  ***Arabidopsis lyrata^1,2,3^***  *(Aly002688; Aly014809; Aly016712; Aly028161);* ***Arabidopsis thaliana^1,2,3^***  *(AT2G33880*  *AT3G03660*  *AT5G17810*  *AT5G45980*  *AT5G46010)* | ***Arabidopsis lyrata^1,2,3^***  *(Aly015918; Aly027052; Aly029745);*  ***Arabidopsis thaliana^1,2,3^***  *(AT1G20700 AT1G20710*  *AT4G35550);*  ***Arachis hypogaea^3^***  *(Ahy002590);*  ***Artemisia annua^3^***  *(Aan003821);*  ***Brassica napus^1,2,3^***  *(Bna002896; Bna006212; Bna018146);*  ***Brassica rapa^1,3^***  *(Bra001224; Bra004033);*  ***Carica papaya^1,2,3^***  *(Cpa003844; Cpa019809);*  ***Citrus sinensis^1,2,3^***  *(Csi002594; Csi006573; Csi009198; Csi011569);*  ***Cucumis sativus^1,2,3^***  *(Csg002899; Csg005213; Csg009511);*  ***Glycine max^1,2,3^***  *(Glyma02g10410*  *Glyma04g01830*  *Glyma06g01940*  *Glyma10g43580*  *Glyma18g52490*  *Glyma20g23220);*  ***Gossypium hirsutum^1,3^***  *(Ghi006162; Ghi006185);* ***Helianthus annuus^3^***  *(Han002587);*  ***Lotus japonicas^1,2,3^***  *(Lja014335; Lja014933; Lja017235; Lja026877);*  ***Malus x domestica^3^***  *(Mdo008276; Mdo013211);* ***Manihot esculenta^1,2,3^***  *(Mes018967; Mes028632; Mes044258);*  ***Medicago truncatula^1,2,3^***  *(Medtr7g025190);*  ***Mimulus guttatus^1,2,3^***  *(Mgu005008);*  ***Nicotiana tabacun^3^***  *(Nta013065);*  ***Populus trichocarpa^1,2,3^***  *(POPTR 0005s27410);*  ***Prunus persica^1,2,3^***  *(Ppe001631; Ppe018604);* ***Raphanus sativus^1,3^***  *(Rsa006061; Rsa007058);*  ***Ricinus communis^1,2,3^***  *(Rco29830t000069*  *Rco30170t000801);*  ***Solanum lycopersicum^1,2,3^***  *(Sly006834);*  ***Solanum tuberosum^1,3^***  *(Stu004033);*  ***Theobroma cacao^3^***  *(Tca001557);*  ***Vigna unguiculata^1,3^***  *(Vun003266; Vun003357; Vun004930; Vun011016);*  ***Vitis vinifera^1,2,3^***  *(Vvi010049; Vvi022239; Vvi044308; Vvi045340; Vvi045475; Vvi045702);* ***Brachypodium distachyon^1,2,3^***  *(Bdi002646; Bdi012336);*  ***Hordeum vulgare^1,2,3^***  *(Hvu014144);*  ***Lycoris longituba^3^***  *(ADG57873);*  ***Oryza sativa^1,2,3^***  *(LOC Os01g60270);*  ***Panicum virgatum^1,2,3^***  *(Pvi007746; Pvi008059);* ***Sacchayum officinarum^3^***  *(Sof004312);*  ***Sorghum bicolor^1,2,3^***  *(Sb03g038080);*  ***Triticum aestivum^1,2,3^***  *(Tae001397);*  ***Zea mays^1,2,3^***  *(GRMZM2G038252*  *GRMZM2G069274*  *AC2189723_FG005);*  ***Amborella trichopoda^3^***  *(CAT02918);*  ***Ginkgo biloba^3^***  *(CAT02909);*  ***Gentum gnemon^3^***  *(CAT02930);*  ***Picea sitchensis^1,3^***  *(Psi002276);*  ***Pinus sylvestris^3^***  *(CAT02934);*  ***Pinus taeda^2,3^***  *(Pta008477);*  ***Selaginella moellendorffii^2,3^***  *(Smo005187; Smo009110; Smo010320; Smo011376; Smo025928; Smo028853);*  ***Physcomitrella patens^3^***  *(Ppa005400; Ppa021887; Ppa024530;)*;  ***Micromonas^3^***  *(Mpu001196; Mpu001866; Mpu007958; Mpu009604;Mrc002433);*  ***Ostreococcus^3^***  *(Olu006328; Orc005963)* |

**Supplementary Table 2: Each clade contains the numbers of species.**

The WUS clade contained 166 sequences within 30 species; the intermediate clade contained 88 sequences within 28 species and the ancient clade contained 96 sequences with in 47 species. The superscripts 1, 2, 3 refer to the species within the WUS clade, the intermediate clade and the ancient clade, respectively.

| Motif No. | Sequences (Name) |
| --- | --- |
| Motif 1 | HD domain ([Gehring et al., 1994](#_ENREF_5)) |
| Motif 2 | MTDEQYETLRKQIAIYGTICERLVEMH |
| Motif 3 | RKRRRLEEAAASASSSPAFSAIATTAAASSGSEQSHHQSYADQIVNDGSFLPDACLFETNVTLEQPRI |
| Motif 4 | EQLHHPAAQSYAAAQIVIDGGVVSIIDDKSPFDLVGHFGDG |
| Motif 5 | VPVDESGVTVEPLQQGAVYIV |
| Motif 6 | LEIKALHVHHRSQRNNTNISSEPAPPRPV |
| Motif 7 | SSNRHWPSMFRSKHATQPWQT |
| Motif 8 | WUS box (Zhang et al., 2010) |

**Supplementary Table 3: Sequences of each motif in WOX from model plants including green algae, *Physcomitrella patens, Selaginella moellendorffii, Pinus taeda,* rice and *Arabidopsis thaliana*.**

**Supplementary Figures**

**
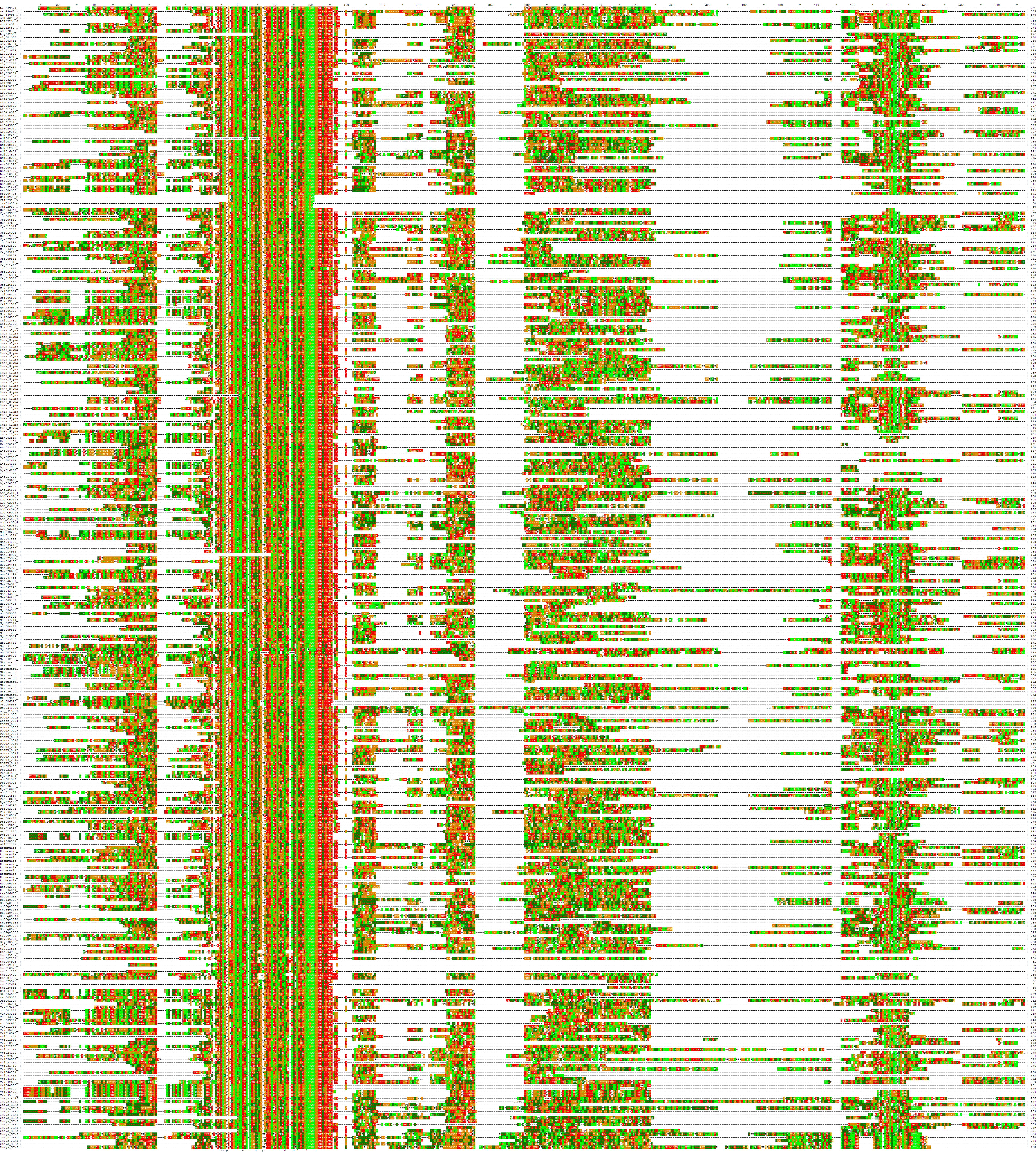
**

**Supplementary Figure 1: Alignment of full-length sequences of 350 WOXs.**

The alignments, which had 350 sequences were obtained using MUSCLE (Edgar 2004). The detail was shown in Supplementary file 1.


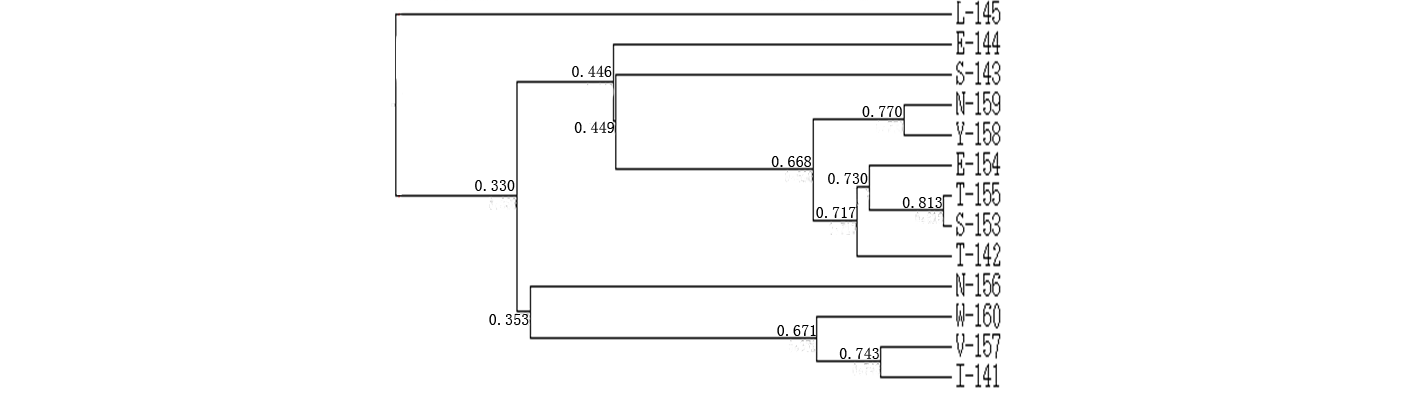


**Supplementary Figure 2: Pairwise positional correlation estimation of WOX proteins.**

The pairwise positional correlation estimation was analyzed by CRASP (http://wwwmgs.bionet.nsc.ru/mgs/programs/crasp/). The pairwise positions (indicated by number in the figure) from 141 (I) to 145 (L) and from 153 (E) to 160 (W) in the homeodomain are correlated in WOX family.


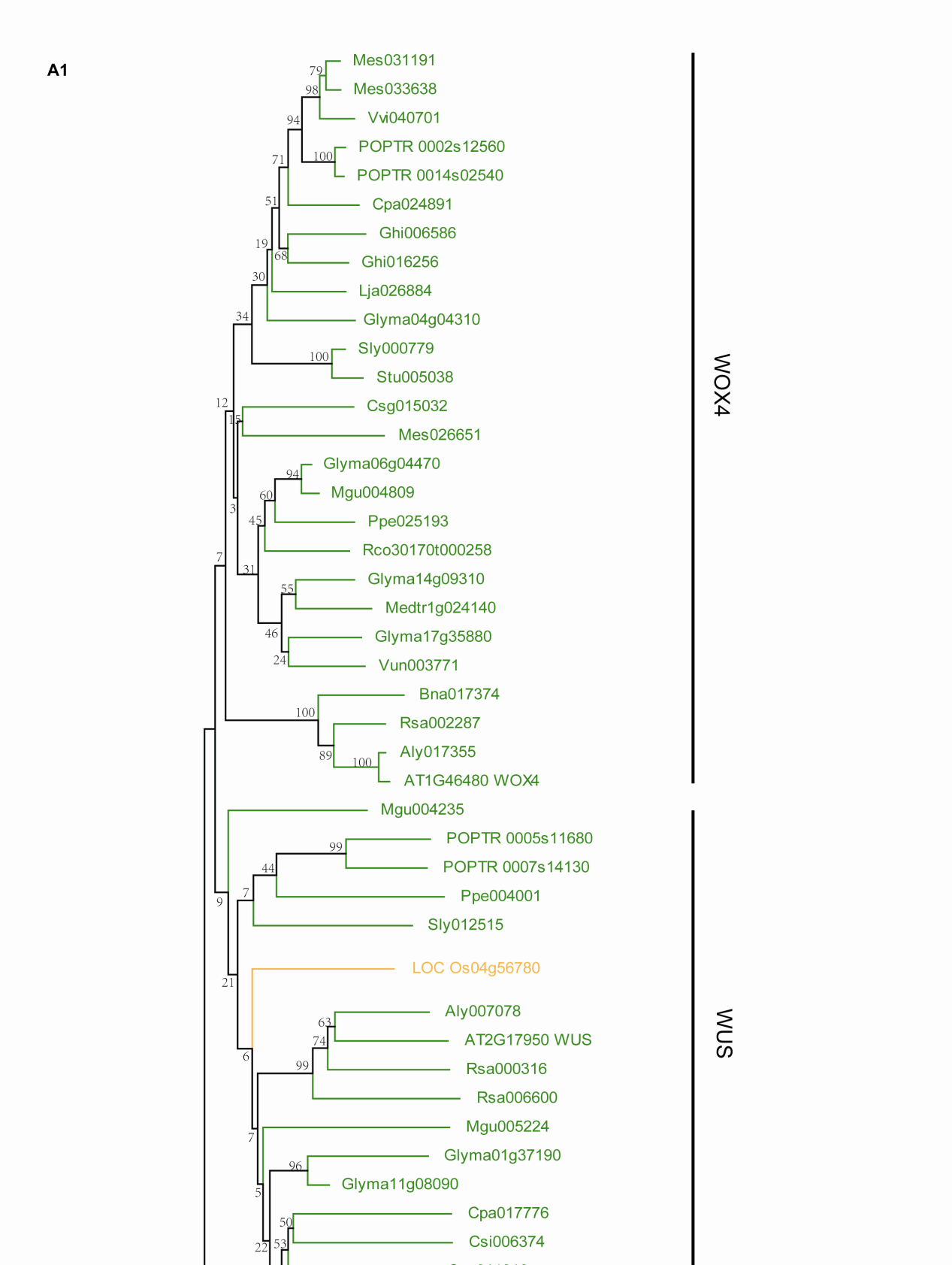

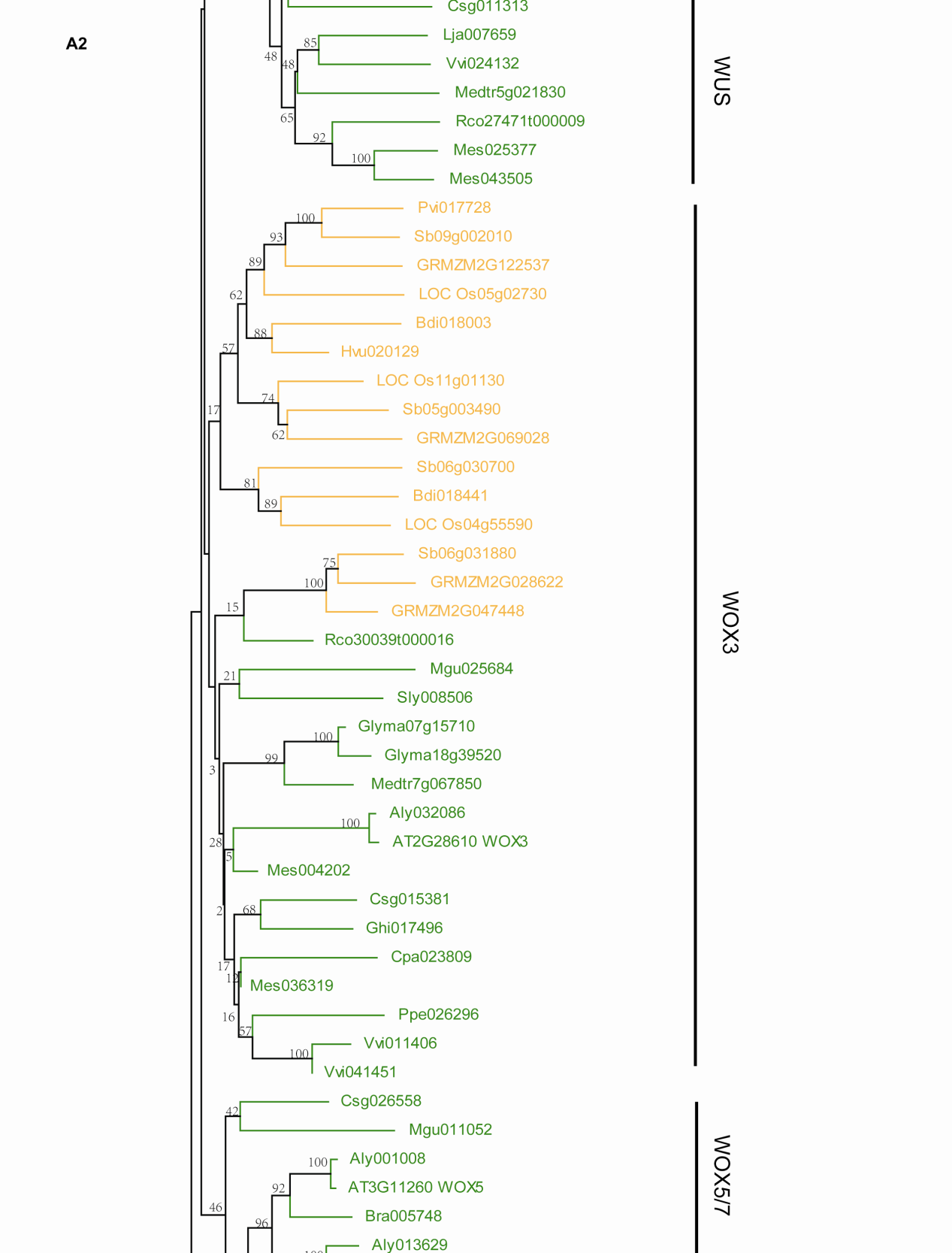

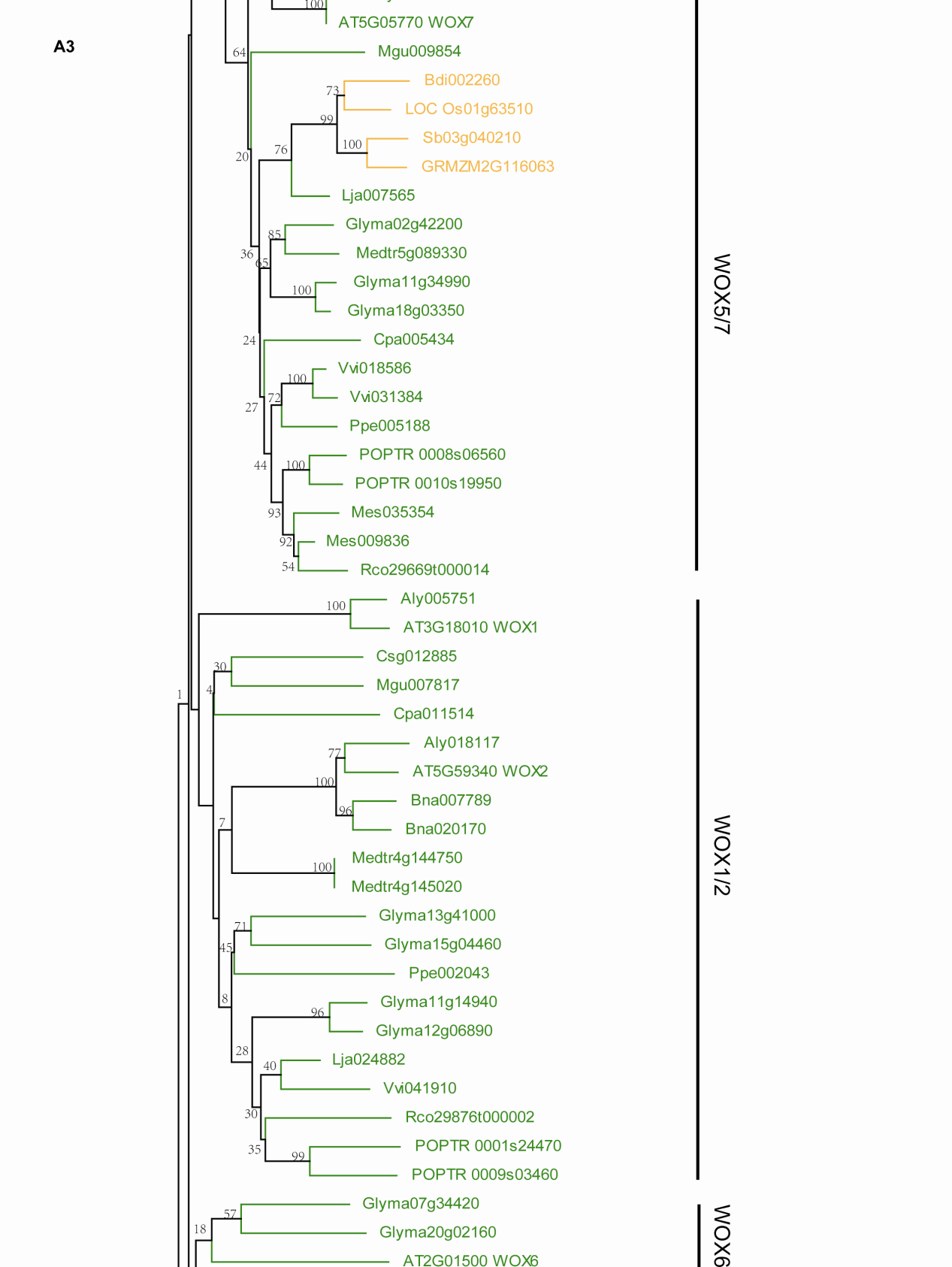

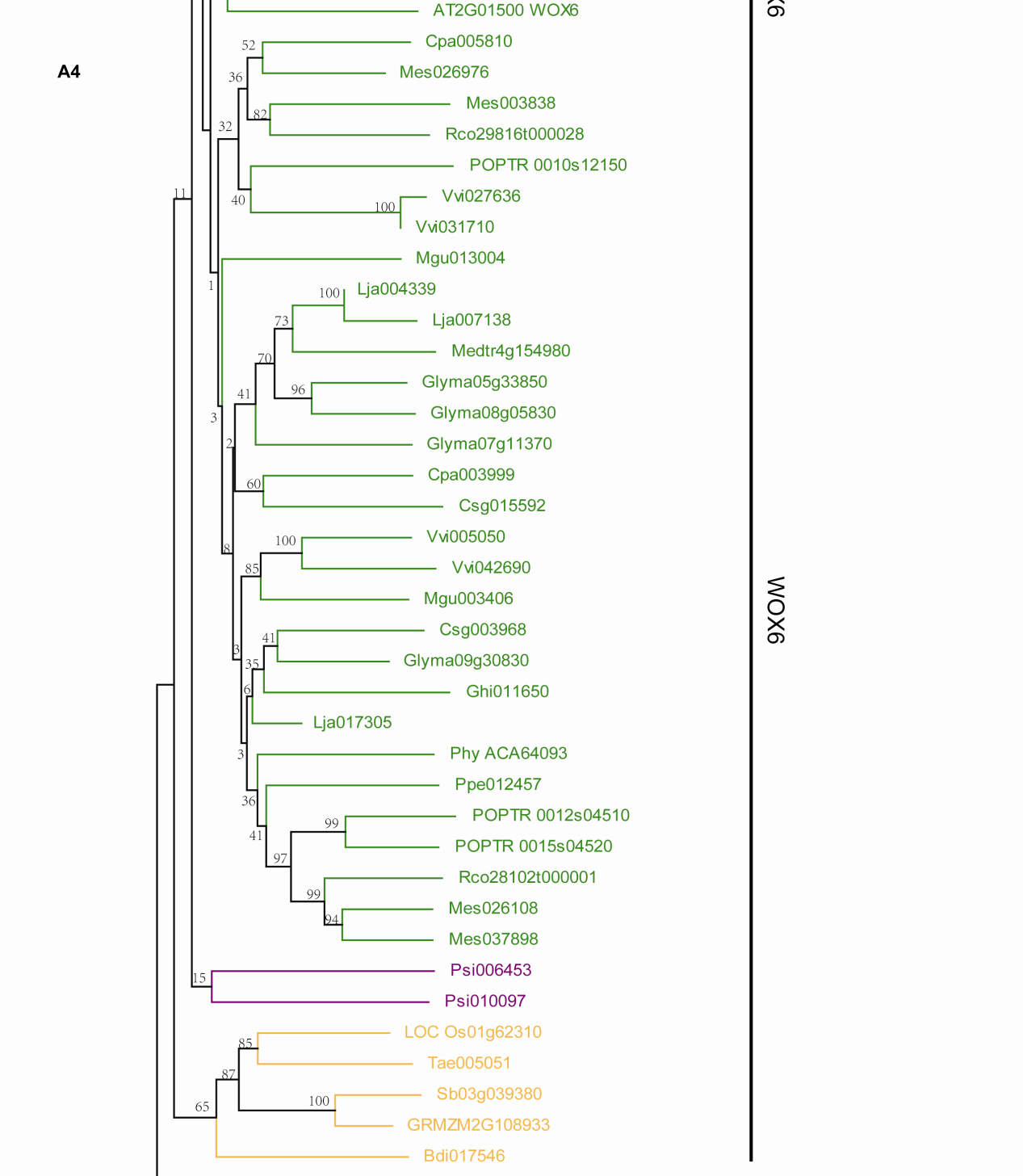

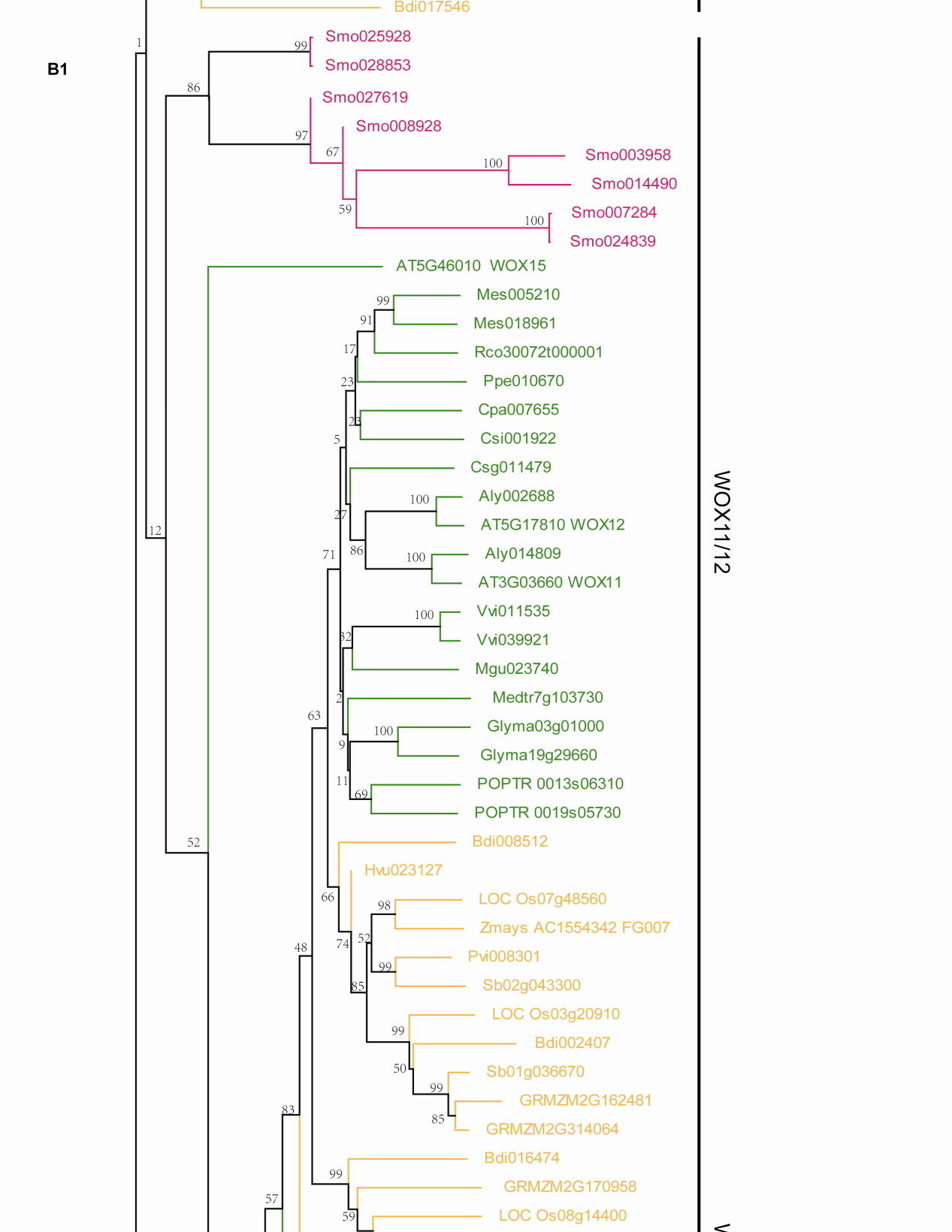

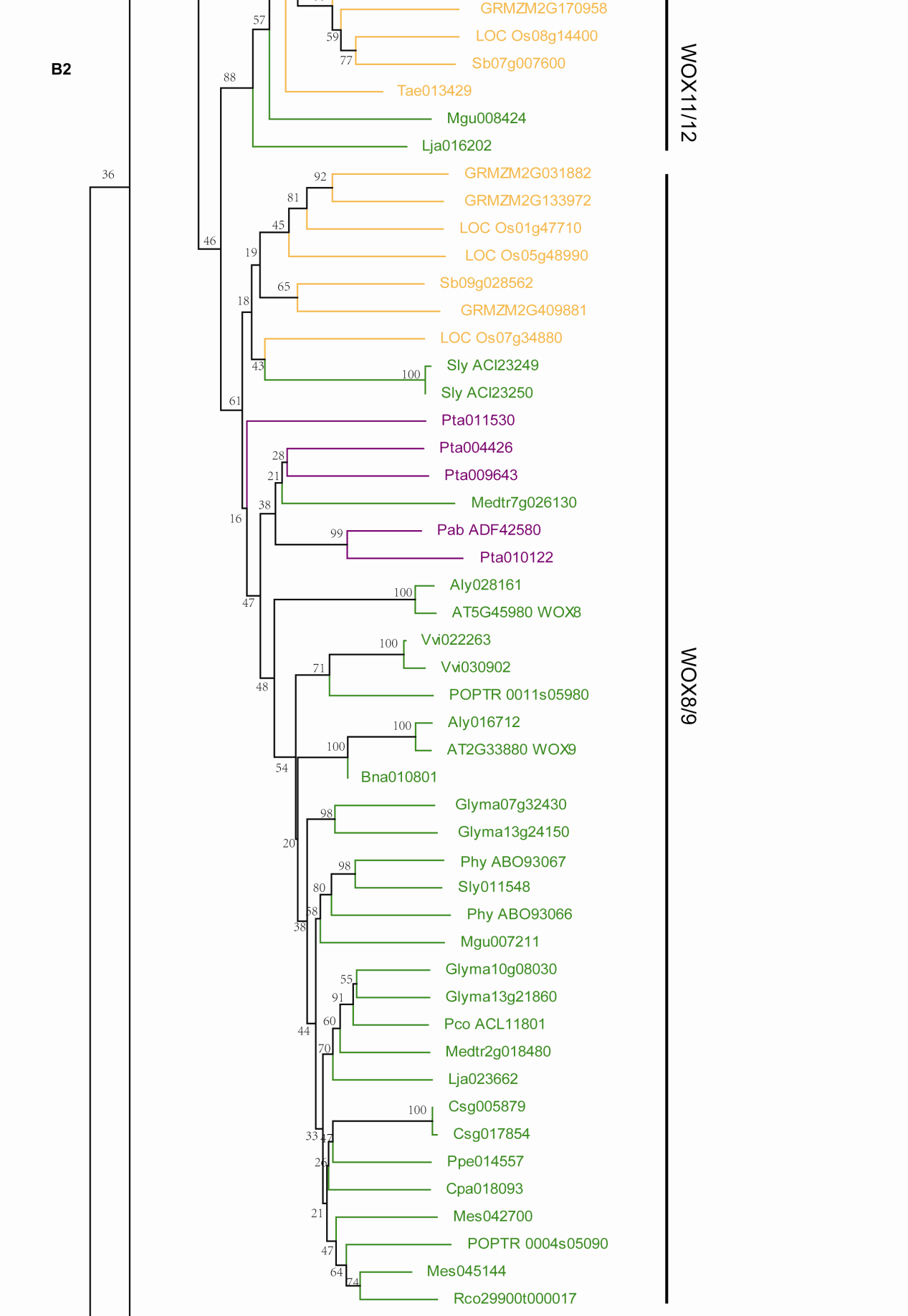

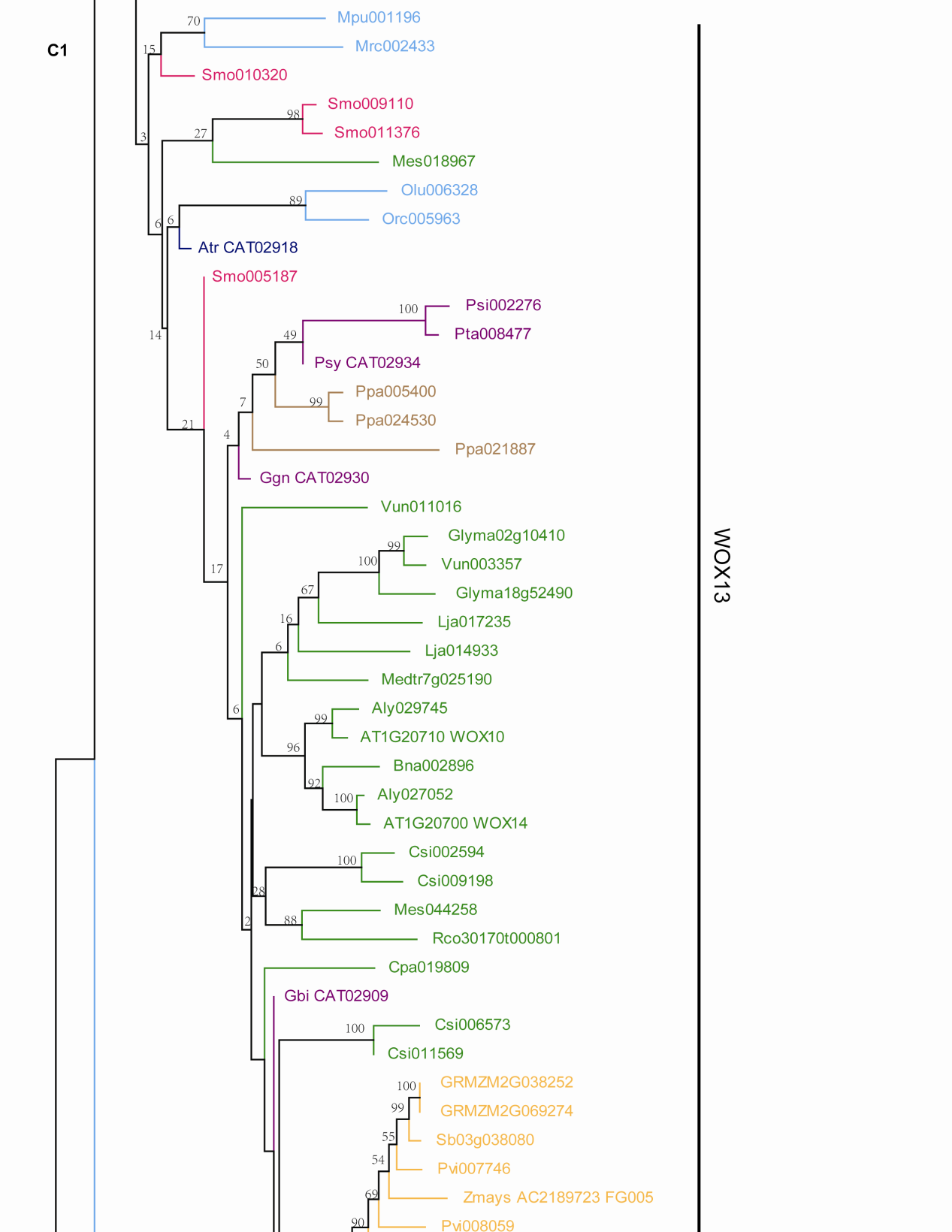

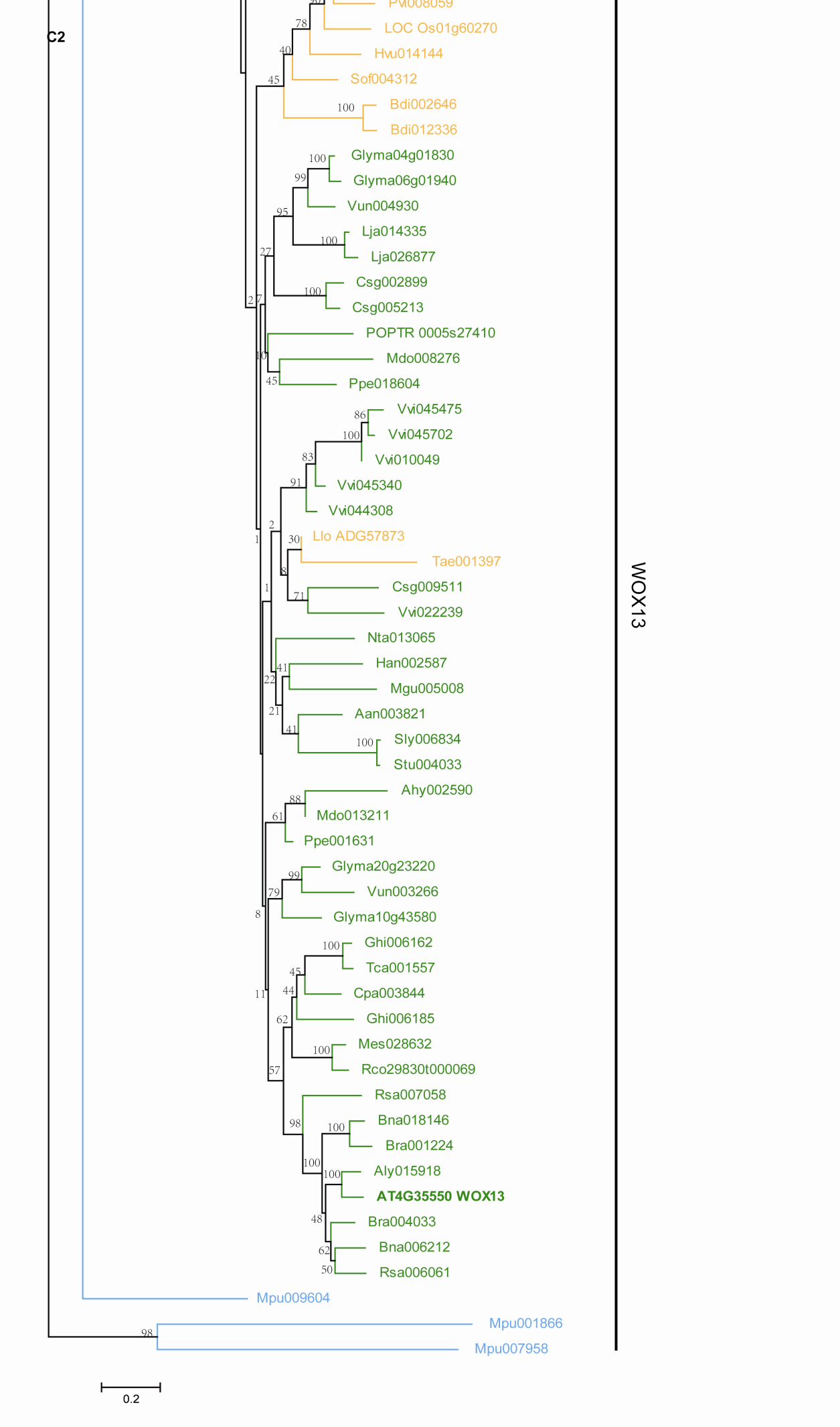


**Supplementary Figure 3: The full phylogeny of WOX family.**

The phylogenetic tree, constructed by MAGE 4 (Kumar et al. 2004) using 350 sequences of proteins from 50 species from green algae to angiosperms, is the neighbor joining (NJ) tree. This tree had 3 subclades, A is WUS clade, which contains 166 sequences, 30 species; B is intermediate clade, which contains 88 sequences, 28 species and C is ancient clade, which contains 96 sequences, 47 species. Green algae, bryophyta, lycopodiophyta, gymnosperm, *Amborella trichopoda,* monocots and eudicots colored wathet blue, garnet, dull red, violet, dark blue, orange and green, respectively. Mes: *Manihot esculenta* Vvi: *Vitis vinifera* POPTR: *Populus trichocarpa* Cpa: *Carica papaya* Ghi: *Gossypium hirsutum* Lja: *Lotus japonicus* Glyma: *Glycine max* Sly: *Solanum lycopersicum* Csg: *Cucumis sativus* Mgu: *Mimulus guttatus* Ppe: *Prunuspersica* Rco:*Ricinus communis* Medtr:*Medicago truncatula* Vun: *Vigna unguiculata* Bna:*Brassica napus* Rsa: *Raphanus sativus* Aly: *Arabidopsis lyrata* Csi:*Citrus sinensis* Pvi: *Panicum virgatum* Sb:*Sorghum bicolor* Bdi:*Brachypo diumdistachyon* Hvu: *Hordeum vulgare* GRMZM: *Zea mays* LOCOS: *Oryza sativa* Olu (Orc): *Ostreococcus* Bra:*Brassica rapa* AT: *Arabidopsis thaliana* Psi: *Picea sitchensis* Tae: *Triticum aestivum* Smo: *Selaginella moellendorffii* Pta: *Pinus taeda* Pab: *Picea abies* Phy: *Petunia x hybrida* Pco: *Phaseolus coccineus* Mpu (Mrc): *Micromonas* Atr: *Amborella trichopoda* Psy: *Pinus sylvestris* Ppa: *Physcomitrella patens* Ggn: *Gnetum gnemon* Gbi: *Ginkgo biloba* Sof: *Saccharum officinarum* Mdo: *Malus x domestica* Llo: *Lycoris longituba* Nta: *Nicotiana tabacu* Han: *Helianthus annuus* Aan: *Artemisia annua* Stu: *Solanum tuberosum* Ahy: *Arachis hypogaea* Tca: *Theobroma cacao*.
